# Opportunity Costs or Not? Validating the Short Gambling Harm Screen against a Set of “Unimpeachable” Negative Impacts

**DOI:** 10.3390/jcm10030549

**Published:** 2021-02-02

**Authors:** Cailem Murray Boyle, Matthew Browne, Matthew Rockloff, Tracy Flenady

**Affiliations:** 1 School of Psychology, Central Queensland University, Brisbane 4000, Australia; 2 School of Psychology, Central Queensland University, Bundaberg 4670, Australia; m.browne@cqu.edu.au (M.B.); m.rockloff@cqu.edu.au (M.R.); 3 School of Nursing, Midwifery and Social Sciences, Central Queensland University, Rockhampton 4701, Australia; t.flenady@cqu.edu.au

**Keywords:** gambling-related harm, screen, validity, unimpeachable, gambling harms

## Abstract

Assessing the harmful consequences of gambling is an area of active investigation. One measure intended to capture gambling-related harm is the 10-item short gambling harm screen (SGHS). Although good psychometric properties have been reported, it has been suggested that the screen’s less severe probes may not represent genuinely harmful consequences, but rather may reflect rational opportunity costs. Consequently, it has been argued that the screen may lead to overestimation of the extent of gambling-related harm in the population. The current study sought to examine the psychometric performance of three less severe suspect items in the SGHS. Associations between each of these items and a specially constructed scale of relatively severe “unimpeachable” gambling harms were calculated from archival data from 5551 Australian and New Zealand gamblers. All three suspect items, both individually and upon aggregation, predicted greater endorsement of “unimpeachable” harms, and indicated the presence of gambling problems. Moreover, the SGHS as a whole is highly correlated with “unimpeachable” gambling harms. Including suspect items in the SGHS was found to improve predictions of low- and moderate-risk gambling status, but slightly decreased predictions of severe gambling problems. The results are inconsistent with the notion that SGHS harm probes capture either inconsequential consequences or opportunity costs. They confirm prior findings that harm symptomatology is unidimensional, and that the report of multiple more prevalent, but less severe, harms serves as an effective indicator of the spectrum of experienced harm.

## 1. Introduction

Harm-minimisation is now well established as a framework for developing policy and interventions to address gambling problems. However, the population screens most widely used to capture these problems typically focus on identifying problematic or at-risk gamblers, often incorporating elements of behavioural addiction [1,2,3]. Harm-minimisation, by contrast, recognises a continuum of harm as the primary outcome of interest at the individual level [4,5]. Aggregation at a population-level can identify the spatial or social distribution of impact, as well as tracing the total “burden” of gambling problems over time [6]. This information can be valuable for researchers and public-health experts when developing needs-based resource allocation strategies to optimise harm reduction [7,8]. Measurement of such a broad ranging, subjectively experienced outcome requires a robust tool, able to faithfully capture underlying harm associated with gambling.

### Short Gambling Harm Screen

The short gambling harm screen (SGHS) was designed to capture the degree of gambling-related harm (GRH) experienced by an individual, from very mild to severe [9]. The SGHS has a simple format: respondents answer “Yes” or “No” to 10 binary-scored items. Each item describes a specific gambling-related harm, e.g., “Felt like a failure”. Respondents are prompted to only consider harms they have determined resulted from their gambling, e.g., “Considering the last 12 months, did you experience any of the following as a result of your gambling?”. SGHS items were selected to maximise sensitivity, capturing the broadest spectrum of GRH possible [9]. This sensitivity is important because gamblers experiencing mild degrees of harm are the most prevalent in the population [7]. Previous studies have reported that the SGHS has robust psychometric properties [9]. Additionally, the scale’s binary response format was found to perform at near parity with a four-point Likert alternative [10]. Though the SGHS has since been applied a number of times in the literature [11,12,13,14], several criticisms recently levelled at the SGHS demand attention.

Recent papers [15,16] have argued that the SGHS includes several items which may not describe genuine gambling-related harms. These include three of the most prevalent, and least severe, items selected from Browne et al.’s [17] comprehensive 72-item pool of gambling harms: (1) “Reduction of my available spending money”, (2) “Reduction of my savings”, and (3) “Less spending on recreational expenses, such as eating out, going to movies or other entertainment”. All three items capture non-acute financial impacts argued to represent rational opportunity costs, given that every dollar spent on gambling naturally limits funds available for other purposes [16]. If these items merely reflect rational economic decisions, we expect that their impact on health and wellbeing should be negligible. If they are not indicative of an underlying continuum of harm, they also ought to have little relationship with either gambling problems or any more severe harms.

Given the above, whether particular SGHS items are invalid appears to be a straight-forward question to address. Are certain SGHS probes indicating something other than “genuine” harm resulting from excessive involvement in gambling (e.g., a rational opportunity cost)? If so, we would expect them to: (a) have low coherence with “unimpeachably” severe harms, either in the SGHS or in the broader harms checklist; (b) have a low association with the underlying dimension of harm indicated by the total harms checklist; and (c) have a low to negligible relationship with gambling problems, as measured by the Canadian Problem Gambling Severity Index (PGSI).

## 2. Design

### 2.1. Datasets and Participants

Quantitative analyses of three archival datasets (NZ, VIC, VAL; see Table 1) were conducted to address the research questions. All datasets had been cleaned prior to analysis. Data from all samples were collected within 5 years of the current analyses. Datasets differed somewhat in their inclusion criteria (VAL: gambled in the past 12 months; NZ, VIC: gambled in the past 6 months) as well as geographical range. Despite these differences, all three samples were amalgamated into a combined dataset. This amalgam was used for all analyses in the current study except for those conducted for comparator scale development (VAL survey only). Data from a total of 5551 Australian and New Zealand adults were analysed. All participants reported gambling at least once in the past 12 months prior to being surveyed.

### 2.2. Benchmarks

The current design aimed to test the proposition that a subset of SGHS items reflect opportunity costs rather than genuine harms. Three benchmark criterion variables were used: an ad-hoc “unimpeachable” gambling harms scale (UGHS), a latent harm variable calculated from most of the 72 gambling consequences, and the Canadian Problem Gambling Severity Index (PGSI) [1]. We evaluated the SGHS and each of its constituent items with respect to these three benchmarks. Whilst the latent factor is arguably the “gold standard” for the entire dimension of harm (as it ranges from mild to severe) the UGHS, by design, will be weighted towards a more acute level of harm.

#### 2.2.1. Unimpeachable Gambling Harms Scale

Items were drawn from the aforementioned pool of candidate gambling-related harms [17] to create the UGHS. Five criteria were used to select items from the pool. Item face validity (or item content) had a higher priority during this process than would be expected for other more psychometrically robust scales. Item content was analysed by subjective appraisal of the candidate harms. Each candidate item, having satisfied criteria 1 and 2 below, required consensus among the authors that they depicted an obviously harmful circumstance. This process removed 16 items from consideration. Some degree of subjectivity in this item-selection process was unavoidable as the criticisms being addressed are grounded in a similar subjectivity. Item content was supplemented with comparisons to PGSI scores as a measure of construct validity. While the PGSI is an imperfect proxy for harm, there is a consensus in the literature that most moderate-risk and problem gamblers are harmed.

UGHS items were selected contingent upon meeting the following criteria: (1) not among the 10 items included in the SGHS; (2) appropriate construct validity, as defined by significant correlation with PGSI (*p* < 0.05); (3) face validity, i.e., “unimpeachably harmful”; (4) equal representation of items from each harms category where achievable (given a priori application of criteria 1–3); and (5) high prevalence, where there was sufficient response frequency in the sample for statistical power (*n* ≥ 30). In total, 10 items were selected from six different harms categories (see Table 2). All items were significantly associated with the PGSI (*p* < 0.001) and had a prevalence greater than 30 (Min. *n* = 46). The minimum prevalence (minimum percentage endorsed) of each item among the three datasets was greater than the average prevalence of the respective category of harm for 7 out of the 10 items (see Table 2).

#### 2.2.2. Latent Harm Variable

One way to describe the purpose of the SGHS is to serve as a brief surrogate for the principle latent factor of all gambling harms. Therefore, in addition to the UGHS, a variable capturing latent harm was created using items in the gambling harms pool [17]. Previous gambling theory and empirical work suggests that both gambling problems and harms lie on a continuum of unidimensionality among these items, collectively providing broad construct coverage [17,20]. The pool contains a relatively high proportion of “unimpeachably” severe harms (e.g., increased experience of depression, loss of significant assets), and is generally regarded as comprehensive. Thus, the latent factor of all harms in this checklist should well-capture the theoretical construct GRH, and serve as a valuable benchmark for assessing suspect SGHS items. We estimated the latent variable associated with the total set of gambling harms (g_h_) in the current study. A confirmatory factor analysis (CFA) of 68 of the 72 items was conducted to estimate the g_h_ score for each respondent. 

#### 2.2.3. Problem Gambling Severity Index

The PGSI is intended to capture gambling problems, rather than harm. However, it is a widely used and rigorously validated measure of gambling problems, and half the items describe negative impacts [1,21]. It was thus considered an obvious candidate for construct validation. Scores from the PGSI (between 0 and 27), reflect the degree of problem gambling risk (0: Non-Problem Gambler (NPG); 1–2: Low Risk (LR); 3–6: Medium Risk (MR); 7+ Problem Gambler (PG)) [1].

### 2.3. Statistical Analysis

#### 2.3.1. Reliability and Internal Structure

Coefficient omega (ω) was used rather than Cronbach’s alpha (α) to measure scale reliabilities of the SGHS and UGHS. Coefficient ω is interpreted similarly to α, on a scale from 0 to 1. However, ω is a more stringent test of internal coherence, capturing the degree to which a scale is unidimensional as well as reliable. Unlike α, coefficient ω reliability estimates do not require that: (1) items are loaded (unstandardized) onto the factor equally; and (2) item error variances are uncorrelated. These assumptions often lead to α overestimating reliability in real-world data [22]. 

A Rasch analysis was conducted to capture discrimination and difficulty thresholds for each SGHS item across all three datasets (see Appendix A).

#### 2.3.2. Performance of SGHS as Predictor of Harm

Bivariate correlations were conducted between the SGHS and criterion measures to test whether SGHS predicted “genuine” harms. Should several items not represent “genuine” harms, we expected SGHS and UGHS scales to be associated either weakly or not at all. Furthermore, as measures of “unimpeachable” harms, we expected both the UGHS and PGSI to be better associated with the latent variable g_h_ than the SGHS was. To test this, we conducted differential analyses of bivariate correlations between these scales (see [23] for methodology). 

#### 2.3.3. Performance of SGHS as a Classifier of Problem Gambling

In addition to inter-scale correlations, analyses of the sensitivities and specificities of the SGHS and the UGHS were conducted. The objective of these analyses was to determine how effectively the screens discriminated between: (a) NPGs (PGSI = 0) and LR gamblers or higher (LR+; PGSI ≥ 1); (b) NPG and LR gamblers (PGSI < 3) and MR gamblers or higher (MR+; PGSI ≥ 3); and (c) NPG, LR, and MR gamblers (PGSI < 7) and PGs (PGSI ≥ 7). The classification performance of the SGHS and UGHS in predicting PGSI risk category were compared using the area under the receiver operator curve (AUC). DeLong’s method [24] was used to test whether any differences in AUC between the two screens were due to different discriminatory properties or chance.

#### 2.3.4. Performance of “Milder” SGHS Items as Predictors of Harm

To check whether suspect SGHS items ‘Reduction of my available spending money’ (Spend), ‘Reduction of my Savings’ (Sav), and ‘Less spending on recreation’ (Rec) had a determinantal effect on the total SGHS performance, these items were removed from the screen. Scale bivariate correlations with all three criteria (UGHS, PGSI, g_h_) were subsequently revisited. Pearson values before and after item removal were compared to determine any change in magnitude or direction [23].

#### 2.3.5. Performance of SGHS as a Predictor of Problem Gambling Categorisation

Descriptive breakdowns of the percentage of respondents in each PGSI risk category at each score on the SGHS were tabulated to determine change in category composition as scores increased. This process was repeated with only “milder” items included (1–3).

## 3. Results

### 3.1. Reliability and Internal Structure

Both the SGHS (ω = 0.85) and the UGHS (ω = 0.85) possessed identical and high internal coherence. Rasch-model parameter estimates indicated that all SGHS items reported high (>1) discrimination in all three datasets, except for “Sold personal items” and “Increased credit card debt”, in the VIC sample only (see Appendix A). Notably, none of the suspect items were poor discriminators, however they were among the items with the lowest severity thresholds in all three datasets.

### 3.2. Performance of SGHS as Predictor of Harm

Table 3 shows the SGHS performed strongly as a predictor of harms across a range of measures. The screen was strongly positively correlated with the UGHS (r = 0.73), as well as the PGSI (r = 0.68) and the latent variable gh (r = 0.87). The PGSI was also associated with the latent variable gh (r = 0.79), though less strongly than the SGHS was (Δr = −0.08, z = −16.9, *p* < 0.001). However, it should be borne in mind that that latent factor is derived from the original 72-item checklist, which includes all SGHS and UHS items. Thus, it is legitimate to use this benchmark to compare individual items with each other, and to compare the relative performance of the SGHS and UHS, but not to compare these scales with the PGSI.

### 3.3. Performance of SGHS as a Classifier of Problem Gambling

**Figure 1:**
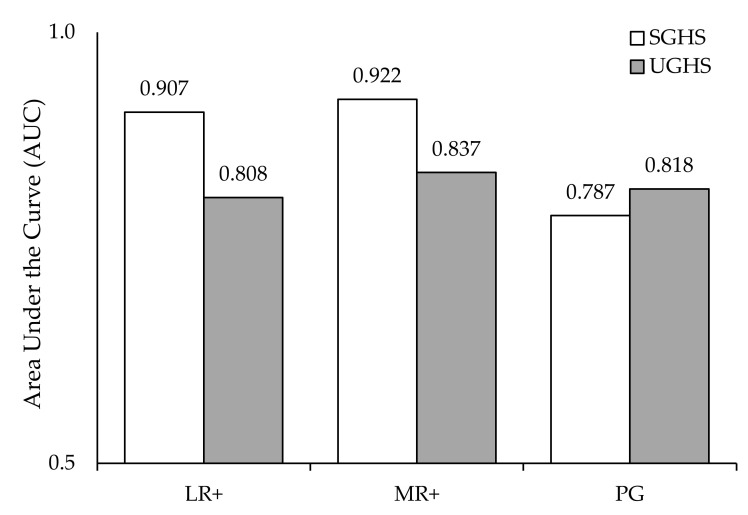
Areas under the curve when classifying respondents across different PGSI category thresholds.

Consistent with previous findings, the SGHS reliably detected at-risk or problematic gamblers (Sensitivity = 88%; Specificity = 84%). Figure 1 demonstrates differences between the SGHS and UGSH when discriminating between different PGSI categorisations. When discriminating between NPGs and LR+, both the SGHS (AUC = 0.907) and the UGHS (AUC = 0.808) performed above-chance (0.5). Using DeLong’s method for comparing AUCs, the SGHS demonstrated superior discrimination to the UGHS when differentiating NPGs and L+ (z = 22.84, *p* < 0.001). 

Similarly, when the screens were used to classify respondents as either NPG/LR or MR/PG (MR+; PGSI ≥3), both the SGHS (AUC = 0.922) and UGHS (AUC = 0.837) performed well. The SGHS again discriminated with greater fidelity than the UGHS in detecting MR+ (z = 19.37, *p* < 0.001). When classifying gamblers into either PG or non-PG (NPG, LR, or MR) status, the SGHS (AUC = 0.787) discriminated slightly less effectively than the UGHS (AUC = 0.818; z = −4.62, *p* < 0.001).

### 3.4. Performance of “Milder” SGHS Items as Predictors of Harm

#### 3.4.1. Association of Individual SGHS Items with Benchmarks

Table 4 summarises the relationship of each SGHS item with respect to each of the three benchmarks. All individual SGHS items were positively correlated with the UGHS, including the three suspect items (Spend: r = 0.39, Sav: r = 0.40, Rec: r = 0.43; *p* < 0.001). Each suspect item was also positively associated with both PGSI scores (Spend: r = 0.36, Sav: r = 0.42, Rec: r = 0.34; *p* < 0.001), and g_h_ (Spend: r = 0.42, Sav: r = 0.46, Rec: r = 0.45; *p* < 0.001). 

#### 3.4.2. Analysis of Correlation Coefficient Differentials

Removal of “milder” items (1–3) from the SGHS led to a mild increase in association strength with both the UGHS (Δr = 0.02, z = 7.11, *p* < 0.001) and PGSI (Δr = 0.01, z = 3.27, *p* = 0.001). However, the strength of the relationship between the SGHS and the g_h_ weakened by a greater magnitude when these items were removed (Δr = −0.03, z = −12.54, *p* < 0.001). 

### 3.5. Performance of SGHS as a Predictor of Problem Gambling Categorisation

**Figure 2:**
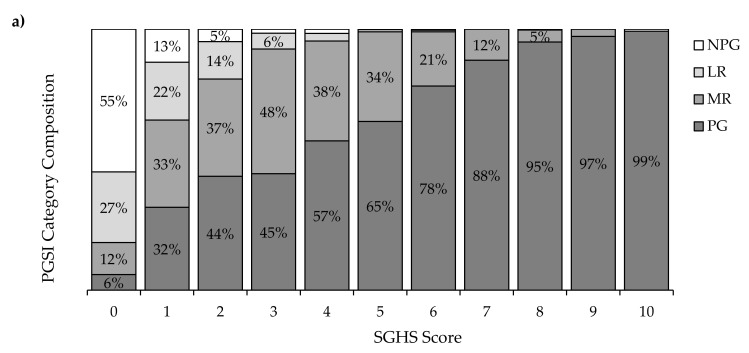

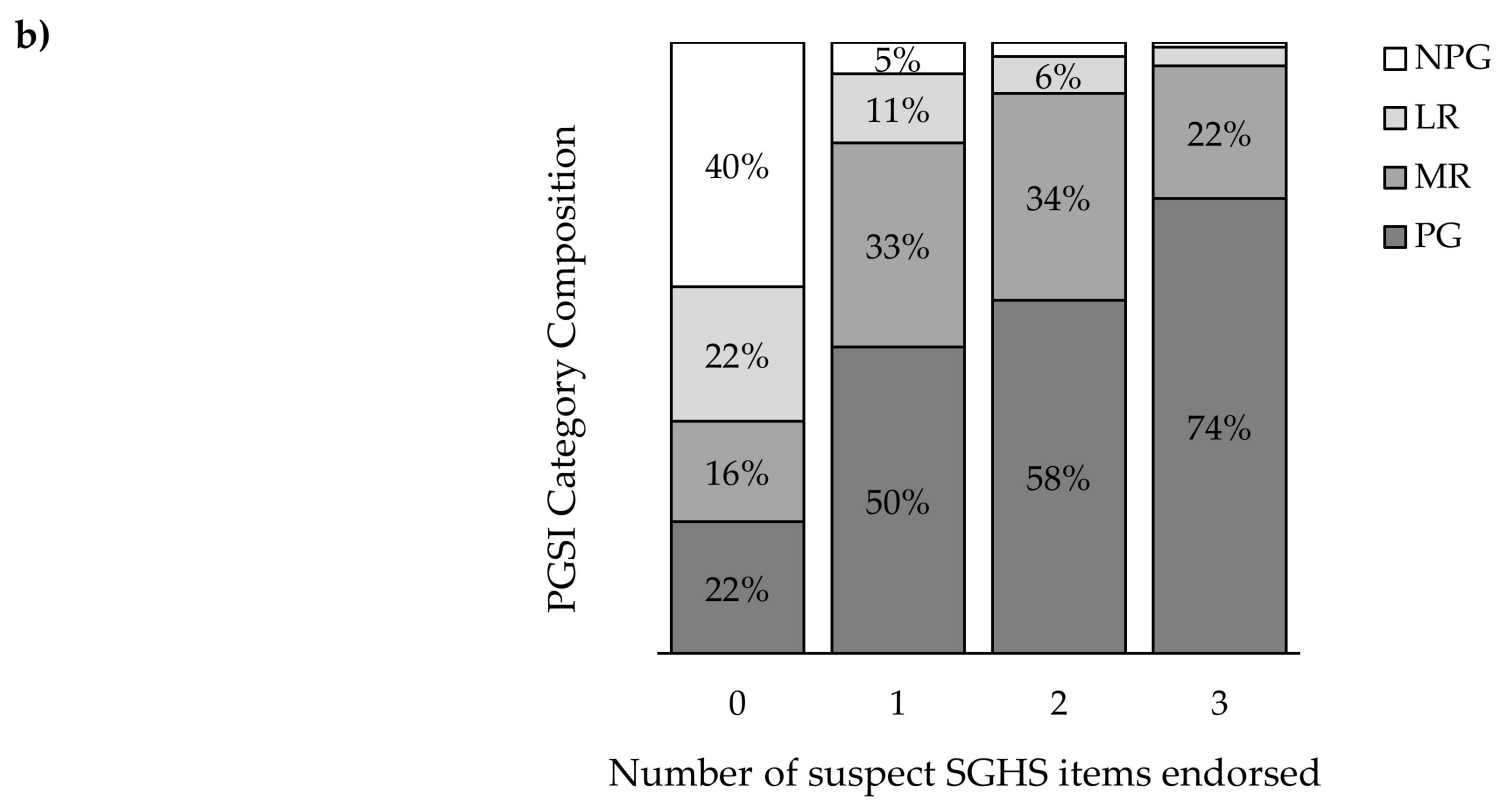
Breakdown of PGSI category among gamblers by: (**a**) SGHS score (top), (**b**) number of “milder” SGHS items endorsed (bottom).

Figure 2a shows the changing composition of PGSI-categorised respondents with respect to each score of the SGHS (0–10). As shown in the figure, the probability of a respondent being classified as a PG increases approximately linearly in the range of SGHS scores between 1–9. This change is particularly marked between SGHS = 0 and SGHS = 1 (ΔPG (0,1) = 26%). When checking suspect items independently to other SGHS items, per-item increases in PG prevalence were similarly steep (ΔPG (0,1) = 28%, see Figure 2b). Three-quarters (74%) of those endorsing all 3 suspect items were PGs.

## 4. Discussion

The current study re-analysed existing survey data to test the reliability and construct validity of the SGHS, as well as to address concerns that several constituent items do not depict meaningful harm. The high coefficient omega reliability supported prior results regarding the scale’s reliability and unidimensionality. A lower omega coefficient would have been expected if the three constituent items were capturing a factor other than harm, such as opportunity costs of gambling.

We found that SGHS scores were not only strongly related to g_h_ scores, but were more closely aligned with the g_h_ than the UGHS. We checked whether scores on the SGHS could be used to accurately classify respondents into PGSI categories. The SGHS yielded good classification of individuals at different levels of risk for gambling problems. It was much superior to the UGHS for moderate degrees of gambling problems. The UGHS was slightly better at classifying respondents in the PG category, which would be expected given our selection of items that captured more serious consequences. Consistent with this, the prevalence of gambling problems increases steadily with increasing SGHS score (Figure 2a). Somewhat strikingly, even the subset of suspect items provided a strong indication of risk for gambling problems, with 74% of individuals who responded positively to all three items being categorised as problem gamblers. Furthermore, a single suspect item in isolation was a significant predictor of PGSI and UHS scores, as well as the latent harm factor. Taken together, these results appear to comprehensively contradict the notion that the SGHS includes items that do not indicate genuine gambling-related harm.

A more nuanced finding of the present study is that the suspect items, individually, do indeed better capture milder degrees of harm, rather than tapping the more severe end of the spectrum. The magnitude of the relationship of the SGHS with both the UGHS and PGSI marginally increased when suspect items were removed. Nevertheless, the relationship between the SGHS and g_h_ decreased after suspect items were removed. This is consistent with the original intent of the SGHS, which was to capture the entire spectrum of gambling-related harm; “milder” items afford the SGHS broader coverage of the total spectrum of harm, rather than only capturing severe gambling consequences, or the most severe degree of problem gambling risk.

## 5. Conclusions

The proposition that the SGHS contains invalid items that do not indicate genuine gambling-related harm implies that these items should have low or nil association with gambling problems and “unimpeachable” harms. It also implies that removal of suspect items should improve the general functioning of the scale. Our analysis did not support any of these predictions. The original SGHS appears to be an effective indicator of the spectrum of gambling-related harm.

## Figures and Tables

**Table 1:** Datasets and average scores on the Short Gambling Harm Screen (SGHS) and Problem Gambling Severity Index (PGSI).

Abbr.	Dataset/Study	Sample (*n*)	Male (%)	Age ($\bar{x}$)	SGHS ($\bar{x}$ *[s]*)	PGSI ($\bar{x}$ *[s]*)
NZ	Measuring the Burden of Gambling Harm in New Zealand. (2017) [18]	951	57.4%	-	3.6 *(2.7)*	7.5 *(5.7)*
VIC	Victorian population gambling and health study (2020) [19]	3076	55.7%	*-*	4.4 *(2.7)*	10.1 *(6)*
VAL	Validation of a short screen for gambling related harm (SGHS): A tool for assessment of harms from gambling (2017) [9]	1524	49.4%	45	1.2 (*1.9)*	3.8 *(5.4)*

**Table 2:** “Unimpeachable” gambling harms scale (UGHS) items. Category of harm, minimum single-sample prevalence, and Pearson’s correlations (r) with PGSI scores are shown.

#	Harm	Category	Min Prev. (%)	Min Prev. (*n*)	PGSI (r)
(1)	Late payments on bills	Financial	7.4%	113	0.42 *
(2)	Less spending on essential expenses	Financial	4.6%	70	0.41 *
(3)	Social isolation	Relationships	4.0% ^†^	61	0.40 *
(4)	Greater tension in my relationships	Relationships	6.6%	101	0.46 *
(5)	Felt insecure or vulnerable	Psychological	7.3% ^†^	111	0.46 *
(6)	Felt worthless	Psychological	7.6% ^†^	116	0.47 *
(7)	Loss of sleep due to time gambling	Health	9.1%	139	0.42 *
(8)	Did not eat as much/often as I should	Health	5.6%	85	0.35 *
(9)	Was absent from work or study	Work/Study	4.7%	72	0.35 *
(10)	Did not fully attend to children	Deviance	3.0%	46	0.22 *

^†^ Items did not have a prevalence greater than category median. * *p* < 0.001.

**Table 3:** Scale correlation matrix. Confidence intervals (95%) shown in parentheses.

Scale	SGHS (7 Items)	UGHS	PGSI	g_h_
SGHS (10 items)	0.95 * *(0.94–0.95)*	0.73 * *(0.71–0.75)*	0.68 * *(0.70–0.65)*	0.87 * *(0.86–0.88)*
SGHS (7 Items)	-	0.75 * *(0.73–0.77)*	0.69 * *(0.67–0.72)*	0.84 * (0.84–0.86)
UGHS	-	-	0.68 * *(0.70–0.65)*	0.85 * *(0.83–0.86)*
PGSI	-	-	-	0.79 * *(0.78–0.81)*

* *p* < 0.001.

**Table 4:** Evaluation of individual SGHS items with respect to three benchmarks. Suspect items shaded grey.

#	SGHS Item	Cat.	UGHS	PGSI	g_h_
1	Reduction of my available spending money *(Spend)*	Fin.	0.39 *	0.36 *	0.54 *
2	Reduction of my savings *(Sav)*	Fin.	0.40 *	0.42 *	0.55 *
3	Less spending on recreational expenses *(Rec)*	Fin.	0.43 *	0.34 *	0.52 *
4	Had regrets that made me feel sorry about my gambling	Psy.	0.44 *	0.38 *	0.52 *
5	Felt ashamed of my gambling	Psy.	0.47 *	0.48 *	0.58 *
6	Sold personal items	Fin.	0.50 *	0.43 *	0.49 *
7	Increased credit card debt	Fin.	0.39 *	0.41 *	0.46 *
8	Spent less time with people I care about	Rel.	0.51 *	0.45 *	0.58 *
9	Felt distressed about my gambling	Psy.	0.49 *	0.48 *	0.58 *
10	Felt like a failure	Psy.	0.58 *	0.49 *	0.60 *

* *p* < 0.001.

## Data Availability

Data available upon request; please direct enquiries to the lead author.

## Appendix A

**Table A1:** Rasch model (IRT) discrimination (β) and difficulty (α) values SGHS items in each sample.

SGHS Item	VIC		NZ		DEV	
	β (θ)	α	β (θ)	α	β (θ)	α
Spend	1.74	−0.60	1.57	−0.58	2.14	1.01
Savings	1.34	−0.31	1.51	0.14	2.69	1.13
Recreation	1.64	0.01	1.23	0.13	1.82	1.46
Regret	1.74	0.01	1.69	0.27	1.96	1.62
Shame	1.59	0.06	1.86	0.43	2.45	1.41
Items	0.87 ^†^	1.79	1.32	1.60	1.70	2.22
Credit Card	0.75 ^†^	1.12	1.05	1.39	2.04	1.77
Time	1.12	0.40	1.68	0.61	2.54	1.56
Distress	1.78	0.30	2.48	0.64	3.25	1.47
Failure	1.13	0.65	1.54	1.06	2.65	1.56

^†^ below discrimination threshold (>1).
